# Readiness for prehospital point-of-care ultrasound among EMS personnel in a system where ultrasound is not yet implemented: a national cross-sectional survey

**DOI:** 10.1186/s12873-026-01594-9

**Published:** 2026-04-27

**Authors:** Ertuğ Günsoy, Fatma Selman, Behnan Gülünay, Ahmet Aykut, Cem Yıldırım, Çağlar Kuas

**Affiliations:** 1 Emergency Department, SBU. Van Education and Research Hospital, Van, Türkiye; 2 Emergency Department, Koç University Hospital, İstanbul, Turkey; 3 Sivas State Hospital, Sivas, Turkey; 4 Department of Emergency Medicine, College of Medicine and Health Sciences, Eskisehir Osmangazi University, Eskisehir, 26350 Turkey

**Keywords:** Prehospital care, Point-of-care ultrasound, Emergency medical services, Readiness, Training, Implementation

## Abstract

**Background:**

Point-of-care ultrasound (POCUS) is increasingly used in emergency and critical care for rapid, radiation-free bedside imaging. However, its integration into prehospital emergency medical services (EMS) remains inconsistent, particularly in paramedic-based systems where physician support is limited. This study evaluated the knowledge, attitudes, and readiness of EMS personnel toward adopting POCUS in a national prehospital system where ultrasound has not yet been implemented.

**Methods:**

A descriptive, cross-sectional survey was conducted between March and August 2023 among prehospital EMS professionals working in Türkiye’s national emergency medical services system, where prehospital ultrasound is not routinely implemented. A 27-item online questionnaire assessed demographics, previous ultrasound training and use, perceived diagnostic applications, and barriers to field implementation. Quantitative data were analyzed descriptively and compared using chi-square tests, while qualitative responses underwent thematic analysis.

**Results:**

A total of 156 EMS professionals participated, most being paramedics (73.1%) working in urban settings (58.3%). Only 21.2% had prior ultrasound training and 22.4% hands-on experience. Nevertheless, 95.5% expressed willingness to receive training, and 90.4% reported interest in using POCUS during prehospital care. The most prioritized applications were intra-abdominal hemorrhage (96.2%), pneumothorax (82.7%), aortic aneurysm (85.3%), and cardiac tamponade (80.1%). Key barriers included lack of equipment, limited training opportunities, and lack of clear regulatory guidelines and defined scope of practice for EMS personnel regarding ultrasound use.

**Conclusion:**

EMS personnel in a paramedic-based prehospital system demonstrated high readiness but limited exposure to POCUS. Structured training programs, pilot implementation projects, and clear regulatory frameworks may support the safe and effective integration of ultrasound into prehospital emergency care.

## Introduction

Point-of-care ultrasound (POCUS) has become an indispensable tool in emergency and critical care for its ability to provide rapid, radiation-free, bedside imaging that supports time-critical decision-making [1–3]. Over the past decade, its use has extended into the prehospital environment, where early ultrasound assessment can guide triage, inform interventions, and improve transport decisions in trauma, cardiac arrest, and other life-threatening conditions [4–6].

The extent to which prehospital POCUS has been adopted varies greatly worldwide, reflecting differences in service organization, staffing models, and regulatory frameworks. In several European countries such as Germany, France, and Norway, physician-staffed ambulance systems have incorporated ultrasound into standard prehospital protocols [7]. Conversely, paramedic-based systems in regions such as North America and Australasia have demonstrated feasibility following structured training programs that enable non-physician providers to perform focused examinations safely and effectively [8, 9]. In Türkiye, prehospital emergency medical services (EMS) are delivered mainly through 112 ambulance teams and are predominantly staffed by paramedics and emergency medical technicians (EMTs/ATTs). Standard emergency ambulances typically operate with a three-person crew, commonly composed of paramedics and/or EMTs, while physician-staffed ambulance units are also available within the system. These doctor-staffed teams may be dispatched to both routine emergency calls and higher-acuity situations, including cardiac arrest and transport of critically ill patients.

Despite these advances, many paramedic-led EMS systems have yet to introduce ultrasound into routine prehospital operations. In such settings, readiness, awareness, and perceived barriers among frontline personnel remain largely unexamined. Establishing this baseline is critical for designing targeted education, defining scope of practice, and planning pilot implementations that ensure safe adoption.

Therefore, this cross-sectional study aimed to evaluate the knowledge, attitudes, and readiness of emergency medical services (EMS) personnel toward POCUS within a paramedic-based prehospital system where ultrasound has not yet been implemented, and to identify perceived barriers, educational needs, and potential enablers for its implementation.

## Methods

### Study design and setting

In Türkiye, prehospital emergency medical services (EMS) are primarily delivered by paramedics and emergency medical technicians (EMTs), who typically complete two-year associate degree or vocational training programs, with limited involvement of physicians (general practitioners); however, ultrasound use is not currently part of routine prehospital practice. This study was designed to assess the knowledge, attitudes, and readiness of prehospital healthcare providers regarding the use of ultrasound in this setting. This was a cross-sectional, descriptive survey study targeting personnel working in Türkiye’s EMS. Data were collected online via Google Forms between March and August 2023, and participation was voluntary.

### Participants and sampling

The target population of the study consisted of healthcare personnel actively working in EMS. All active prehospital EMS personnel were eligible for inclusion regardless of their educational background, years of experience, or scope of practice. As EMS in Türkiye primarily consists of paramedics and emergency medical technicians (EMTs), with limited involvement of physicians, the study population reflects this variability in training and clinical responsibilities. A convenience sampling method was used. The survey link was distributed via social media groups and email to reach relevant professionals, and only individuals who voluntarily agreed to participate were included in the study. Inclusion criteria were active duty in prehospital emergency services and full completion of the questionnaire. Incomplete or inconsistent responses were excluded from the analysis.

The minimum required sample size was calculated to be 138 participants, assuming a 95% confidence level, 5% margin of error, and a response distribution of 50%. This calculation was based on standard formulas used in power analysis software such as G*Power. Accordingly, the final sample of 156 respondents was deemed statistically sufficient.

### Data collection tools and procedure

A structured questionnaire developed by the researchers based on a literature review and expert opinions was used as the data collection tool. The questionnaire consisted of 27 items and included both closed- and open-ended questions designed to evaluate participants’ sociodemographic characteristics, work settings, and ambulance types, as well as their knowledge, experience, and attitudes toward the use of ultrasound. The online version of the questionnaire was created using the Google Forms platform and distributed via email and social media groups to personnel working in EMS. The introduction to the form explained the study’s purpose, confidentiality principles, voluntary participation, and that participants could withdraw from the study at any time before submitting their responses. Completion of the questionnaire was considered to imply informed consent. The questionnaire was developed and administered in Turkish, the native language of the participants.

The variables included in the questionnaire were categorized into three main domains aligned with the study objectives: (1) sociodemographic and professional characteristics of the participants (age, gender, professional role, years of experience, work setting, and type of EMS station); (2) knowledge, experience, and training status related to ultrasound use (prior training, previous use, awareness of field use, willingness to receive training and use ultrasound); and (3) potential use of ultrasound in diagnostic and interventional contexts (evaluated through specific pathologies and procedures). Most variables were assessed using closed-ended questions. In addition, two open-ended questions were included to explore participants’ general thoughts and perceived barriers regarding ultrasound. Responses to these questions were analyzed using thematic content analysis. Coding was performed by a single researcher; participant responses were reviewed, initial codes were generated, and similar codes were grouped into themes through an iterative process.

The internal consistency of the items assessing perceived needs for prehospital ultrasound use was evaluated using Cronbach’s alpha after data collection. The resulting Cronbach’s alpha coefficient was 0.79, indicating good internal consistency.

### Data analysis

Data were analyzed using the Jamovi statistical software (Version 26.0). Continuous variables were presented as mean ± standard deviation (SD), while categorical variables were summarized as frequency (n) and percentage (%). Pearson’s chi-square test was used to compare categorical variables between urban and rural groups. A p-value of < 0.05 was considered statistically significant. Responses to open-ended questions were analyzed thematically; similar expressions were grouped, main themes were identified, and representative participant quotes were tabulated.

The study was approved by Non-Interventional Clinical Research Ethics Committee of the Biruni University on 21.02.2023 (Approval No: [2023/78 − 14]). The introductory page of the survey clearly informed participants about the purpose of the study, voluntary participation, and the anonymity of responses. Informed consent was considered obtained upon completion of the questionnaire.

## Results

A total of 156 prehospital emergency healthcare professionals participated in this survey study. The mean age of the participants was 29.7 ± 7.1 years (range: 20–49). Of the respondents, 67.9% were female, and 32.1% were male. In terms of professional roles, the majority were paramedics (73.1%), followed by emergency medical technicians (18.6%), nurses (5.8%), and physicians (2.6%). Regarding the duration of experience in emergency medical services, 35.9% of the participants reported more than 10 years of experience, while 26.9% had 1–5 years, 24.4% had 6–10 years, and 12.8% had less than 1 year of experience (Table 1).

**Table 1:** Demographic and occupational characteristics of the participants

Characteristics	*n* (%)
Age (mean ± SD)	29.71 ± 7.10
Female	106 (67.9%)
Male	50 (32.1%)
Paramedic	114 (73.1%)
Emergency Medical Technician (EMT)	29 (18.6%)
Nurse	9 (5.8%)
Physician	4 (2.6%)
EMS work experience	
< 1 year	20 (12.8%)
1–5 years	42 (26.9%)
6–10 years	38 (24.4%)
> 10 years	56 (35.9%)
Location	
Urban (central)	91 (58.3%)
Rural (non-central)	65 (41.7%)
Population of area	
0–9,999	13 (8.3%)
10,000–99,999	41 (26.3%)
100,000–999,999	63 (40.4%)
1,000,000+	39 (25.0%)
Average distance to center (mean ± SD)	21.81 ± 37.16 km

Age is presented as a mean ± standard deviation. Categorical variables are expressed as numbers and percentages

Of the participants, 58.3% reported working in urban (central) areas, while 41.7% worked in rural (non-central) settings. The population of the areas where participants were stationed most frequently ranged between 100,000 and 999,999 (40.4%), followed by 10,000–99,999 (26.3%), 1,000,000 and above (25.0%), and 0–9,999 (8.3%). The average distance from their work location to the nearest hospital was 21.8 ± 37.2 km. In terms of ambulance types, the majority of respondents (94.2%) operated basic emergency ambulances. Most participants (92.9%) indicated that they transported patients by ground vehicles. Regarding transport duration, the most commonly reported time frame was 0–10 min (35.3%), followed by 10–20 min (27.6%), 30–60 min (15.4%), 20–30 min (12.2%), and over 60 min (9.6%) (Table 2).

**Table 2:** Geographic and operational characteristics of participants’ work settings

Ambulance Types and Transport	*n* (%)
Ambulance type	
Basic emergency ambulance	147 (94.2%)
Advanced/equipped ambulance	5 (3.2%)
Air ambulance	3 (1.9%)
Sea ambulance	1 (0.6%)
Transport mode	
Ground transport	145 (92.9%)
Air transport	3 (1.9%)
Sea transport	1 (0.6%)
UMKE (disaster response)	7 (4.5%)
Transport time	
0–10 min	55 (35.3%)
10–20 min	43 (27.6%)
20–30 min	19 (12.2%)
30–60 min	24 (15.4%)
> 60 min	15 (9.6%)

Distribution of ambulance types and transport characteristics reported by participants. “Basic emergency ambulance” refers to standard ambulances equipped for routine emergencies. “Advanced/equipped ambulance” includes vehicles with specialized equipment for critical care. Transport modes are categorized as ground, air, sea, or UMKE (National Medical Rescue Team). Average transport time from the field to the hospital is presented in minutes

Among the participants, 21.2% reported having previously received ultrasound training, and 22.4% had prior experience using ultrasound. Additionally, 35.9% stated that they were aware of the use of ultrasound in the field or during patient transport. Notably, 95.5% of respondents expressed a desire to receive ultrasound training, and 90.4% indicated that they would like to use ultrasound on patients during prehospital care or transport (Table 3).

**Table 3:** Participants’ ultrasound training, prior use, and willingness to use or learn ultrasound (yes/no responses)

Survey item (Yes/No question)	*n* (%)
Previously received ultrasound training	33 (21.2%)
Previously used ultrasound in practice	35 (22.4%)
Aware of ultrasound use in the field/transport	56 (35.9%)
Willing to receive ultrasound training	149 (95.5%)
Willing to use ultrasound during field transport	141 (90.4%)

Participants’ previous experience with ultrasound and their preferences regarding ultrasound education and field application. Values represent the number and percentage of respondents who had received training, used ultrasound in practice, or were aware of its field use. Additionally, participants’ willingness to receive ultrasound training and to utilize ultrasound during prehospital transport is reported

The most commonly reported pathologies that participants intended to diagnose using ultrasound were intra-abdominal hemorrhage (96.2%), aortic aneurysm (85.3%), cardiac tamponade (80.1%), and pneumothorax (82.7%). The intention to use ultrasound for diagnostic purposes was largely similar between urban and rural groups. Although the intention to diagnose pneumothorax was higher among urban respondents (87.9%) compared to those in rural settings (75.4%), this difference was not statistically significant (*p* = 0.068). Among the interventional applications, the most frequently reported use of ultrasound was for confirming endotracheal tube placement following intubation (84.6%) (Table 4). Both urban and rural EMS providers experience challenges in making diagnostic decisions in the prehospital environment. Rural teams typically spend longer time with patients during transport, yet no significant difference was found between the groups in perceived need or willingness to use POCUS (*p* > 0.05).

**Table 4:** Participants’ perceived need for ultrasound to diagnose specific pathologies and guide procedures, stratified by urban vs. rural work location (multiple responses allowed)

Survey item (Multiple response question)	Urban *n* (%)	Rural *n* (%)	Cronbach’s Alpha if Dropped	*p*-value
Diagnosis				
Intra-abdominal hemorrhage	87 (95.6%)	63 (96.9%)	0.792	1.000
Aortic aneurysm	78 (85.7%)	55 (84.6%)	0.775	1.000
Cardiac tamponade	74 (81.3%)	51 (78.5%)	0.771	0.812
Pneumothorax	80 (87.9%)	49 (75.4%)	0.771	0.068
Fracture	49 (53.8%)	31 (47.7%)	0.758	0.551
Pulmonary embolism	60 (65.9%)	35 (53.8%)	0.775	0.174
Non-traumatic cardiac arrest	44 (48.4%)	22 (33.8%)	0.770	0.100
Hemothorax	75 (82.4%)	49 (75.4%)	0.781	0.384
Volume assessment	39 (42.9%)	26 (40.0%)	0.781	0.848
İntervention				
Intravenous access	69 (75.8%)	43 (66.2%)	0.797	0.253
Intraosseous access	57 (62.6%)	32 (49.2%)	0.785	0.133
Endotracheal tube placement confirmation	80 (87.9%)	52 (80.0%)	0.778	0.260
Triage in mass casualty or disaster settings	60 (65.9%)	41 (63.1%)	0.786	0.843

Comparison of participants’ intention to diagnose specific pathologies using ultrasound between urban and rural work locations. Values are presented as numbers and percentages of respondents in each group. Statistical significance was assessed using the chi-square test of independence. A p-value less than 0.05 was considered statistically significant

A thematic analysis of responses to open-ended survey questions revealed that participants’ general attitudes toward learning ultrasound were predominantly positive. The most common theme was the perception that ultrasound use in the field is “necessary” and “beneficial,” as expressed by 31.2% of respondents. A notable portion of participants (10.8%) emphasized that ultrasound could facilitate faster and more accurate diagnoses, reduce mortality, and save time. Additionally, some respondents (5.0%) highlighted the potential contribution of ultrasound to appropriate hospital selection and prehospital triage processes. A substantial number (10.8%) also believed that ultrasound training would enhance their professional development and clinical competence. Although less frequently, several participants raised concerns regarding systemic limitations, including the lack of ultrasound devices in ambulances, insufficient training opportunities, and time constraints, stating that ultrasound implementation “might not be realistic in the current system.” Interestingly, 47.3% of the responses consisted of brief or unclear statements such as “positive” or “I’d like to,” which were categorized as neutral or vague. In summary, most respondents demonstrated a high level of interest in learning ultrasound, indicating strong motivation for its potential application in the prehospital setting (Table 5).

**Table 5:** Thematic analysis of open-ended responses on general attitudes and perceived barriers toward ultrasound use

Theme	Example quote	Prevalence	%
General Thoughts on Learning Ultrasound			
Neutral or unclear	Positive – I’d like to –.	High	47.3
Positive attitude & willingness	I think it would be beneficial – Absolutely essential	High	31.2
Rapid and accurate diagnosis	Necessary for rapid diagnosis – Saves time	High	10.8
Personal development & motivation	Makes me feel more competent – Motivates professional growth	Moderate	10.8
Support in hospital selection & triage	Important for referring patients to the right hospital	Moderate	5.0
System-level barriers & realism	There is no time in ambulances – Unrealistic for our country	Low	4.0
Perceived Barriers to Learning Ultrasound			
Lack of equipment	No portable devices – No ultrasound machines in ambulances	High	28.0
Lack of trainers or formal training	Trainers are scarce – Limited course slots	Moderate	18.0
Time constraints & workload	Too busy to attend – No opportunity for practice	Moderate	15.0
Anatomical/knowledge gaps	Weak anatomy background – Easy to forget if not practiced	Moderate	14.0
Legal or bureaucratic limitations	Non-physicians lack authorization – Management not supportive	Moderate	10.0
Skepticism or doubt	No time in the field – Nearby hospitals make it unnecessary	Low	8.0

This table summarizes the thematic analysis of open-ended responses related to participants’ general thoughts on learning ultrasound and the perceived barriers to doing so. Themes are presented separately under two main categories: General Thoughts on Learning Ultrasound and Perceived Barriers to Learning Ultrasound. Each theme is accompanied by a representative quote, a qualitative prevalence label (High, Moderate, Low), and the percentage of total responses that aligned with the theme. Themes are listed in descending order based on their frequency of occurrence. Responses categorized as “Neutral or unclear” refer to brief or vague expressions such as “Positive”, “Maybe”, or no meaningful content

## Discussion

This study found a high level of interest and willingness among EMS personnel in Türkiye to receive training in and use point-of-care ultrasound (POCUS) in the prehospital setting. However, prior training and hands-on experience were limited. Participants most frequently identified life-threatening conditions such as intra-abdominal hemorrhage, pneumothorax, aortic aneurysm, and cardiac tamponade as potential targets for ultrasound use. Key barriers included lack of equipment, limited training opportunities, and system-level constraints.

The results align closely with findings from European and North American literature. In physician-staffed EMS systems across Europe, POCUS is widely utilized in trauma and critical care scenarios. Emergency physicians use their hospital-acquired ultrasound skills in the field to facilitate early diagnosis and treatment. For example, in a landmark German study, Walcher et al. (2006) demonstrated that prehospital FAST exams significantly outperformed physical examination alone in detecting internal bleeding and altered treatment or transport decisions in nearly one-third of trauma cases [7]. This and similar studies affirm that prehospital ultrasound contributes significantly to trauma management decisions [8]. In North America, where EMS systems are typically paramedic-based, the adoption of prehospital ultrasound is more recent and not yet standardized. A 2014 survey revealed that only 4% of EMS systems in North America were using POCUS in the field, although 22% reported plans for future implementation [9]. Nevertheless, pilot studies have shown that paramedics can reliably perform focused exams after short training sessions. In a study by Heegaard et al. (2010), paramedics who received six hours of training successfully performed FAST and aortic assessments without transport delays, and their interpretations were in complete agreement with those of emergency physicians [10]. Similar results have been reported by Kim et al. (2012), who demonstrated that emergency medical technicians achieved high diagnostic accuracy for internal bleeding using FAST exams [11]. Although training models vary in duration and structure, even a few hours of focused instruction can enable prehospital providers to perform basic POCUS applications effectively (Meadley et al., 2017) [12].

Both urban and rural EMS teams face similar challenges in making diagnostic decisions during prehospital care. Rural providers spend longer periods with patients due to extended transport times, yet this did not translate into a higher perceived need for ultrasound. The consistently high motivation observed in both groups indicates a broad awareness of POCUS’s potential benefits across different operational settings. Both groups reported similarly high levels of perceived need for ultrasound use in critical scenarios. Specifically, pathologies such as trauma-related internal bleeding, cardiac tamponade, pneumothorax, and aortic aneurysm were commonly cited across both groups, and no statistically significant differences were found between them (*p* > 0.05) (see Table 4). This suggests that the perceived necessity of POCUS is not limited to areas with long transport times. Rather, even in locations where transport to the hospital is rapid, ultrasound is viewed as a valuable tool. The literature similarly emphasizes the role of POCUS in time-sensitive emergencies such as mass casualty triage and cardiopulmonary arrest, where it facilitates quicker decision-making and destination planning [7, 8]. Studies from Europe and North America likewise show that the demand for POCUS is not confined to remote regions but extends to urban EMS systems where even short transport times do not diminish the clinical utility of field ultrasound [10, 12]. These findings support the notion that prehospital ultrasound is not a luxury tied to geographic remoteness but rather a broadly applicable diagnostic support tool for emergency care in any setting.

In Türkiye, the lack of routine prehospital ultrasound use appears to stem from three major issues: training deficiencies, equipment limitations, and lack of regulatory frameworks. However, international experiences offer strategies to overcome these barriers. In physician-based systems, emergency doctors bring existing skills to the field; in paramedic-based systems, dedicated in-service education and certification programs have bridged the gap. In many countries, focused ultrasound courses have been integrated into paramedic continuing education, enabling them to manage trauma and cardiac emergencies even in the absence of physicians [12]. Equipment limitations have also been mitigated through technological advances. Portable, handheld, and ruggedized ultrasound devices are now suitable for field use, making widespread deployment in ambulances more feasible. Regarding legal and administrative challenges, several countries have developed protocols and expanded paramedic scope of practice under medical oversight, enabling safe and standardized implementation of POCUS in the field.

Based on these findings, several systemic recommendations can be made to support the integration of POCUS into Türkiye’s EMS. First, structured training programs should be developed to meet the strong demand for education. Core ultrasound principles should be included in paramedic training curricula, and in-service certification courses should be offered to active EMS personnel. Second, pilot projects can be launched in selected regions to test the feasibility of field ultrasound, particularly in scenarios involving trauma, cardiac arrest, or suspected aortic emergencies. Third, national guidelines and protocols should be developed to define appropriate indications, procedures, and the scope of practice for paramedics. Fourth, physician supervision should be made available in early implementation phases to ensure accurate image acquisition and interpretation—this could include training by emergency physicians and telemedicine support. Finally, a quality assurance system should be established to review ultrasound findings, monitor performance, and provide constructive feedback. By addressing these components, Türkiye’s EMS can progress toward internationally aligned standards and improve diagnostic accuracy and timeliness in the prehospital phase [13].

## Conclusion

This study demonstrates that EMS personnel in Türkiye are highly motivated to learn and integrate POCUS into clinical practice. Despite low levels of prior training and usage, participants largely recognized the diagnostic value of POCUS for life-threatening conditions such as intra-abdominal hemorrhage, pneumothorax, aortic aneurysm, and cardiac tamponade. The absence of a statistically significant difference in perceived need between urban and rural personnel suggests that POCUS is not only seen as beneficial in areas with long transport times but is considered a valuable diagnostic tool across all prehospital settings. The findings indicate that with structured training programs, pilot implementation projects, and the development of national guidelines, POCUS may be feasibly integrated into Türkiye’s EMS system with appropriate training, resources, and regulatory support. With appropriate institutional support, POCUS may play a critical role in improving diagnostic accuracy and patient care quality in the prehospital environment.

## Limitations

This study has several limitations. First, the use of a convenience sampling method and online data collection limited participation to individuals with internet access and voluntary interest, potentially introducing selection bias. Additionally, the total number of individuals who received the survey link could not be determined, and no comparison between respondents and non-respondents was possible. Second, the data were based on self-reporting, which may be subject to recall bias or social desirability bias. Although the sample size was statistically adequate, it may not fully represent all geographic regions or occupational subgroups within Türkiye’s EMS, and certain regions may have been overrepresented. Additionally, some subgroup analyses were based on relatively small numbers (e.g., physicians), which may limit the generalizability of these findings. The study did not include an objective assessment of ultrasound knowledge or skills, which prevents correlating perceived need with actual competency. The study did not include effect size measures (e.g., Cramér’s V), which may limit the interpretation of the strength of associations. Furthermore, qualitative analysis was conducted by a single researcher without formal assessment of inter-rater reliability, which may introduce subjective bias. Finally, due to its cross-sectional design, the study captures attitudes and experiences at a single point in time and cannot evaluate temporal changes or causal relationships.

## Data Availability

The datasets used and/or analyzed during the current study are available from the corresponding author on reasonable request.

## Abbreviations

POCUS: Point-of-care ultrasound
EMS: Emergency Medical Services
EMT: Emergency Medical Technician
FAST: Focused Assessment with Sonography for Trauma
